# Chronic hyperuricemia impairs blood flow recovery in the ischemic hindlimb through suppression of endothelial progenitor cells

**DOI:** 10.18632/oncotarget.24290

**Published:** 2018-01-22

**Authors:** I-Chun Chen, Chin-Sung Kuo, Chih-Cheng Wu, Hsiao-Ya Tsai, Chih-Pei Lin, Szu-Yuan Li, Ruey-Hsing Chou, Po-Hsun Huang, Jaw-Wen Chen, Shing-Jong Lin

**Affiliations:** ^1^ Institute of Clinical Medicine, National Yang-Ming University, Taipei, Taiwan; ^2^ Division of Endocrinology and Metabolism, Department of Medicine, Taipei Veterans General Hospital, Taipei, Taiwan; ^3^ Cardiovascular Research Center, National Yang-Ming University, Taipei, Taiwan; ^4^ National Tsing-Hua University, Institute of Biomedical Engineering, Hsinchu, Taiwan; ^5^ National Taiwan University Hospital, College of Medicine, Taipei, Taiwan; ^6^ Cardiovascular Center, National Taiwan University Hospital, Hsinchu Branch, Hsinchu, Taiwan; ^7^ Department of Pathology and Laboratory Medicine, Taipei Veterans General Hospital, Taipei, Taiwan; ^8^ Department of Biotechnology and Laboratory Science in Medicine and Institute of Biotechnology in Medicine, National Yang-Ming University, Taipei, Taiwan; ^9^ Division of Nephrology, Department of Medicine, Taipei Veterans General Hospital, Taipei, Taiwan; ^10^ Division of Cardiology, Department of Medicine, Taipei Veterans General Hospital, Taipei, Taiwan; ^11^ Department of Medical Research, Taipei Veterans General Hospital, Taipei, Taiwan; ^12^ Institute and Department of Pharmacology, National Yang-Ming University, Taipei, Taiwan; ^13^ Taipei Medical University, Taipei, Taiwan

**Keywords:** hyperuricemia, endothelial progenitor cell, ischemia, neovascularization

## Abstract

**Objective:**

Chronic hyperuricemia is associated with cardiovascular disease, but its impact on endothelial progenitor cells (EPC) and ischemia-induced neovascularization remains unclear. Herein we investigated whether chronic hyperuricemia could impede blood flow recovery in response to tissue ischemia by suppression of EPC.

**Methods:**

Human EPC were isolated and cultured in a high-level uric acid medium for functional testing. Cell proliferation, nitric oxide (NO) production and apoptosis assay were examined. A chronic hyperuricemia mouse model was established by potassium oxonate treatment and/or a high-level uric acid diet to evaluate the actions of chronic hyperuricemia on ischemia-induced blood flow recovery. After 4 weeks of drug treatment, hindlimb ischemia surgery was performed in the control and hyperuricemia mice. Blood flow recovery was followed up every week before and after ischemic surgery using a laser Doppler Perfusion Imager System. The circulating EPC number in the peripheral blood was determined by flow cytometry (Sca-1^+^/Flk-1^+^).

**Results:**

Incubation with a high-level uric acid medium (10 mg/dL) significantly suppressed EPC proliferation, reduced NO production, and lessened phosphorylation of Akt and eNOS. Moreover, EPC treated with high-level uric acid increased reactive oxygen species production, promoted cellular apoptosis and senescence, and also inhibited EPC tube formation. Four weeks after hindlimb ischemia surgery, the chronic hyperuricemia mice had significantly reduced tissue reperfusion, EPC mobilization, and impaired neovascularization in the ischemic hindlimbs compared with the control mice.

**Conclusions:**

Chronic hyperuricemia impaired blood flow recovery and EPC mobilization in response to tissue ischemia, and these effects could have occurred through suppression of EPC.

## INTRODUCTION

Hyperuricemia is becoming a critical medical problem, and its prevalence and related comorbidities have dramatically increased in past decades [1]. Uric acid (UA) is the final product of purine metabolism, and abnormalities in UA metabolism may cause hyperuricemia, gout, and hyperuricemia-related morbidity [2]. There has been a growing interest in the study of the association between hyperuricemia and cardiovascular disorders, such as hypertension [3], coronary artery disease [4], deep vein thrombosis [5], and chronic kidney diseases [6]. Some studies have reported that hyperuricemia may be associated with cardiovascular disease, independent of traditional risk factors [7, 8], but others have suggested that this association is confounded by the coexistence of cardiovascular risk factors [9]. In addition, an increasing body of evidence suggests elevated serum UA in humans is associated with endothelial dysfunction [10], enhanced systemic inflammation [11], elevated oxidative stress [12], and risk of cardiovascular mortality [13]. As such, a high level of UA should be a crucial mediator associated with reduced vasodilator capacity pro-inflammatory and pro-thrombotic states, and could be an independent risk factor for cardiovascular disease.

Ischemia-induced angiogenesis is a necessary process in wound healing and is a physiological response in ischemic tissues [13]. This process begins with degradation of nonfibrillar collagens in basement membrane followed by migration and proliferation of pre-existing, mature endothelial cells, and then incorporation with circulating endothelial progenitor cells (EPC) in endothelial cells *in situ* [8, 13]. These circulating EPC could be mobilized endogenously when triggered by tissue ischemia or exogenously by cytokine stimulation, and differentiated as endothelial cells [14, 15]. Numerous studies have indicated that an inadequate angiogenic response to ischemia may result in severe tissue damage due to decreased new vessel formation [16, 17]. Patschan et al. previously showed that a transient surge in UA concentration may act as a universal herald of tissue injury, and accelerate the recruitment of EPC after renal ischemia [18]. However, the effect of chronic hyperuricemia on ischemia-induced neovascularization, blood flow recovery and EPC angiogenic functions remains elusive. Therefore, we hypothesized that chronic hyperuricemia can inhibit ischemia-induced neovascularization and reduce blood flow recovery in ischemic tissues through inhibition of EPC angiogenic functions.

## RESULTS

### Cultivation and characterization of human EPC

**Figure 1:**
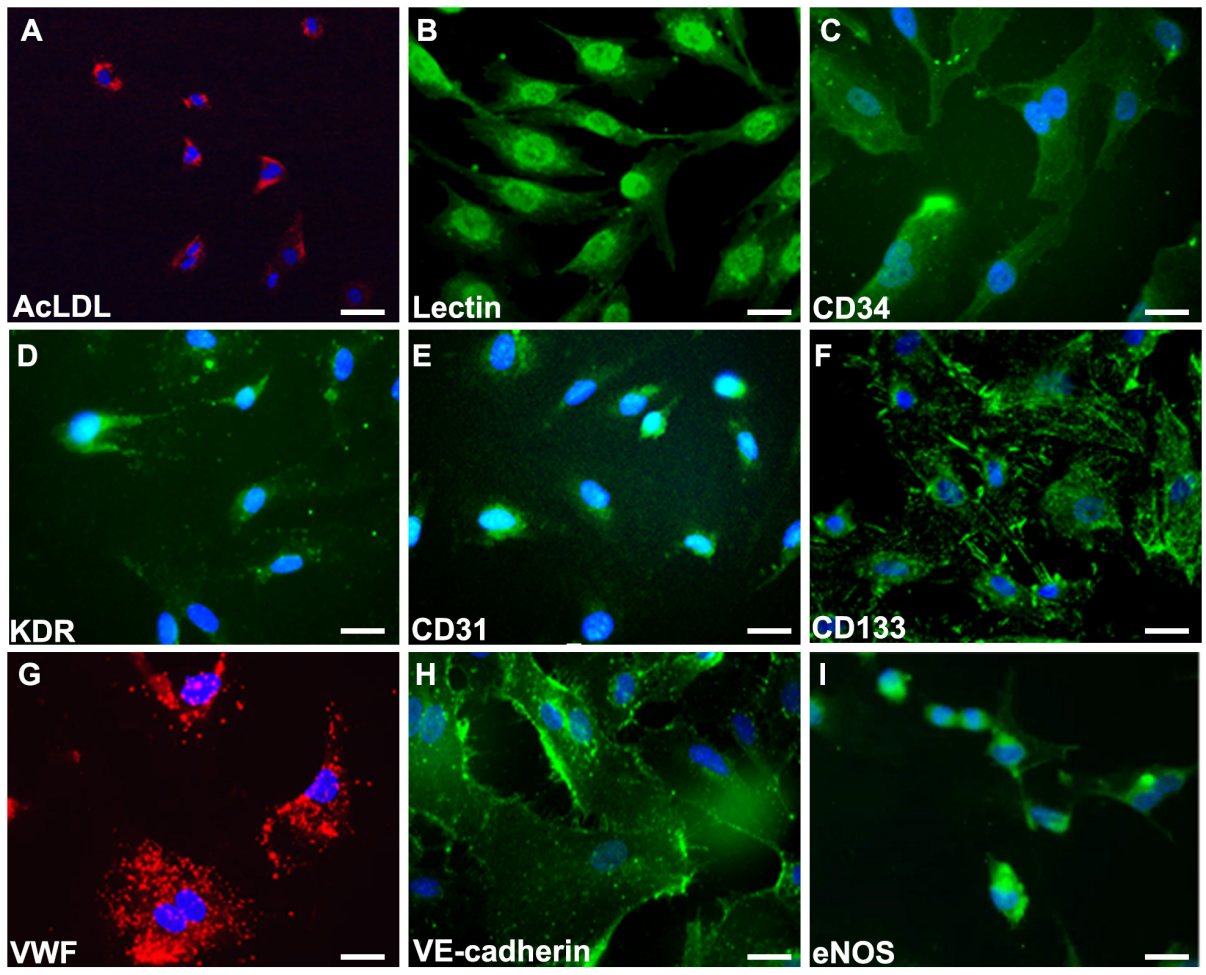
Characterization of late endothelial progenitor cells from peripheral blood Cells was characterized by immunofluorescence detection of **(A)** Dil-AcLDL (red), **(B)** Lectin, **(C)** CD34, **(D)** KDR, **(E)** CD31, **(F)** CD133, **(G)** VWF (red), **(H)** VE-cadherin, and **(I)** eNOS. Cells were counterstained with 4’,6-diamidino-2-phenylindole (DAPI) for nuclear (blue). Scale bar: 50 μm.

As shown in Figure 1, human EPC were isolated from peripheral blood MNCs of healthy adult volunteers. After 4 days of culturing, the medium was changed and non-adherent cells were removed. A certain number of cells continued to grow into colonies of EPC, which emerged 2 to 4 weeks after the start of MNC culture. The EPC exhibited a cobblestone morphology and monolayer growth pattern typical of mature endothelial cells at confluence. EPC were collected and used for all assays in this study. Endothelial cell lineage was further confirmed by indirect immunostaining with the use of 1,1’-dioctadecyl-3,3,3’,3’-tetramethylindocarbocyanine perchlorate-acetylated low-density lipoprotein (DiI-acLDL; Molecule Probe) and co-staining with BS-1 lectin (Sigma) (Figure 1A-1B). EPC characterization was performed by immunofluorescence detection. Most of the cells expressed mature endothelial markers such as CD34, kinase insert domain receptor (KDR), PECAM-1 (CD31), CD133, Von Willebrand factor (VWF), VE-cadherin, and eNOS (Figure 1C-1I), which are considered critical markers of EPC. These images were also performed by quantitative immunofluorescence staining. (Supplementary Figure 1).

### Effects of uric acid on EPC viability and apoptosis

**Figure 2:**
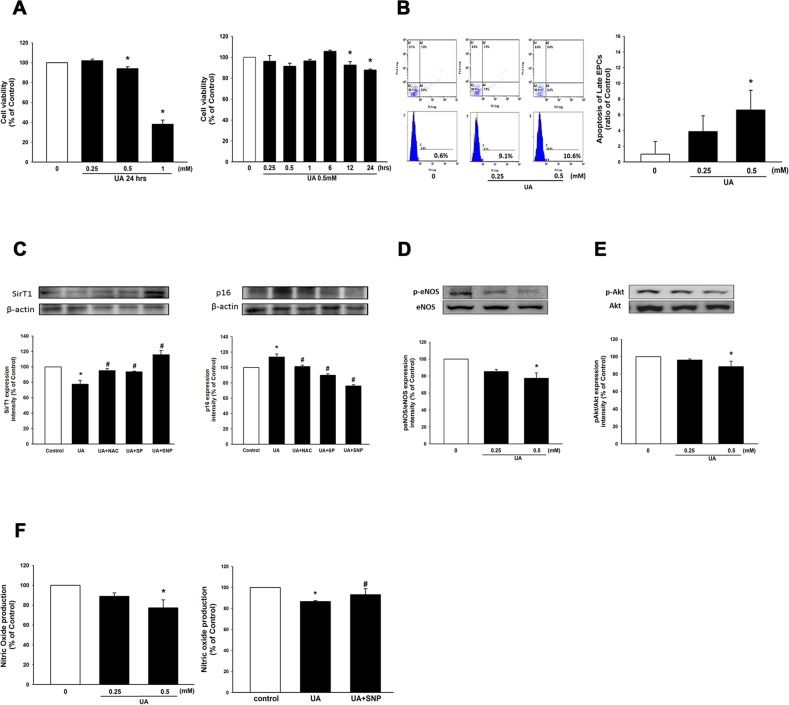
Effects of uric acid on EPC viability, apoptosis, NO production, eNOS and Akt phosphorylation **(A)** EPC viability was analyzed by MTT assay. EPC viability was compared when cultured at different concentrations of uric acid (0, 5, 10, and 20 mg/dL) for 24 hr. and in a high-level uric acid concentration (10 mg/dL) was followed at different time points. **(B)** EPC apoptosis was detected by annexin V assay using flow cytometry. EPC apoptosis was compared at different uric acid concentrations (0, 5, 10 mg/dL). **(C)** Expression of sirT1 and p16 protein levels. **(D)** Expression of phosphorylated eNOS. **(E)** and phosphorylated Akt protein levels. **(F)** NO production was assessed by staining with a NO fluorescent indicator (DAF-FM). NO production was assessed in the high-level uric acid by the presence of SNP. SNP: S-nitroso-N-acetyl-D, L-penicillamine, a NO-donor. Data are mean ± SEM; n=4; ^*^ *P* < 0.05 versus control; ^#^ *P* < 0.05 versus high-level uric acid (UA).

To investigate the effect of hyperuricemia on EPC viability, cultured EPC were treated with high concentrations of UA in dose-dependent and time-dependent manners. As shown in Figure 2A, high-level UA conditions (10mg/dL, 20 mg/dL) significantly reduced EPC viability by 15% and 60%, respectively, when treated for 24 hours. EPC incubated in a high-level UA environment (10mg/dL) for 12 and 24 hours significantly suppressed EPC viability by 7.5% and 12.3%, respectively.

To further confirm this result, cellular apoptotic assay was used to determine the harmful effect of a high-level UA condition on EPC by sorting Annexin V positive cells. As depicted in Figure 2B, administration of UA medium at 5 and 10 mg/dL promoted EPC apoptosis by 3.86±2.31% and 6.62±3.79%, respectively. Moreover, sirT1 and p16 were involved in cellular apoptosis as well. After administration of EPC with a high-level UA medium, sirT1 was significantly downregulated compared to the control, and p16 was upregulated compared to the control. (Figure 2C) These findings suggest that a high concentration of UA induces cellular apoptosis and suppresses EPC proliferation.

### High-level uric acid reduced nitric oxide production and phosphorylation of eNOS and Akt

Hyperuricemia is associated with endothelial dysfunction [10] and high-level UA attenuates eNOS activation in human umbilical vein endothelial cells (HUVEC) [19]. Therefore, we investigated whether a high-level UA condition could impair eNOS activation and decrease NO production in EPC. After administration of EPC with a high-level UA medium, phosphorylation of eNOS and Akt were significantly downregulated compared to the control (Figure 2D-2E). When EPC was treated with a high-level UA medium (10mg/dL) for 24 h, the amount of NO release was significantly reduced compared to the control group. In addition, administration of S-nitroso-N-acetyl-D, L-penicillamine (SNP), a NO-donor, reversed high-level UA-impaired NO production (Figure 2F). These results suggest a high-level UA concentration reduced eNOS/Akt activation and suppressed NO production.

### High-level uric acid stimulates reactive oxygen species production, and activates caspase 3 & 9 proteins and the JNK pathway

**Figure 3:**
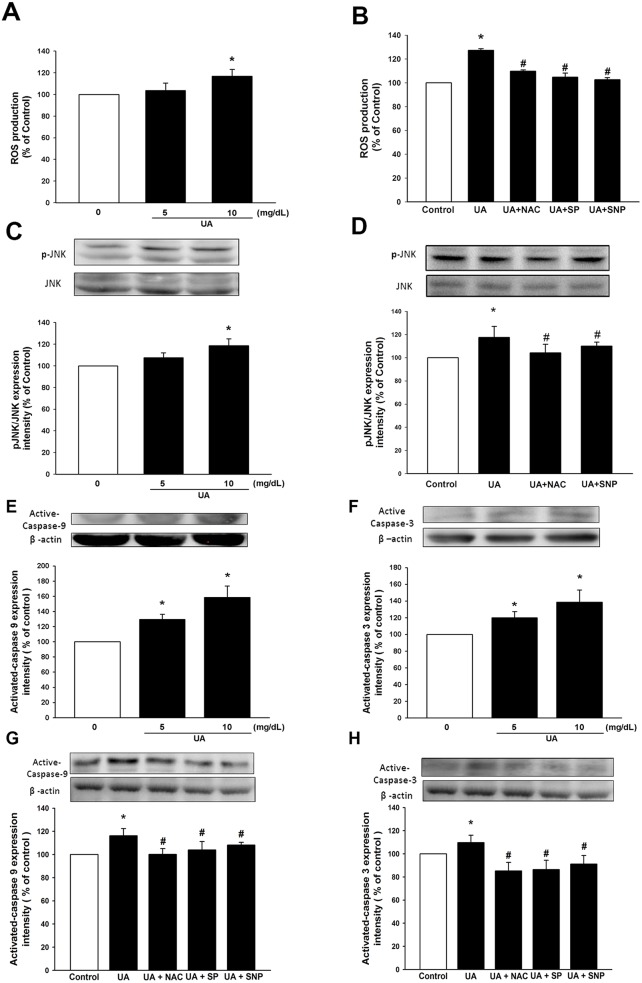
Uric acid induces ROS and active caspase 3, 9 through JNK pathway Cells were cultured and analyzed under different uric acid concentrations (0, 5, 10 mg/dL) and in the absence or presence of SNP (NO donor), NAC (antioxidant) and SP (SP600125, JNK inhibitor). **(A, B)** ROS production of EPC was assessed by staining with DCFH-DA. **(C, D)** Expression of JNK phosphorylation protein levels was assessed by western blot analysis. **(E, F, G, H)** Expression of active caspase-9 and caspase-3 protein levels was assessed by western blot analysis. Data are mean ± SEM; n=4; ^*^ *P* < 0.05 versus control; ^#^ *P* < 0.05 versus high-level uric acid (10 mg/dL).

Oxidative stress is known to be a major mechanism of endothelial dysfunction, and could play a crucial role in high-level UA-induced cellular dysfunction. We explored the effect of high-level UA on ROS production in EPC. As shown in Figure 3A, there was an increase in the generation of ROS after treating EPC with high-level UA. Moreover, this elevation of ROS status could be suppressed by administration of an antioxidant, glutathione precursor (N-acetylcysteine/NAC) (Figure 3B).

In order to elucidate the mechanism of the pathway through which UA induced EPC apoptosis, JNK phosphorylation was determined in EPC treated with a high-level UA medium. As shown in Figure 3C, phosphorylated-JNK expression was enhanced in response to high-level UA stimulation, compared with the control group. The administration of NO-donor (SNP) and antioxidant (NAC) attenuated the activation of UA-stimulated JNK (Figure 3D).

Caspases are a family of protease enzymes that play important roles in programmed cell death and inflammation. We then identified the expressions of caspase 3 and 9 in EPC treated with high-level UA. After treatment of EPC with a high-level UA medium, there was a significantly elevated expression of active-form caspase-9 and 3 (Figure 3E-3F). In addition, administration of an antioxidant and NO donor reversed high-level UA-induced caspase 3 and 9 upregulation (Figure 3G-3H). These effects were also significantly mitigated by administration of JNK inhibitor, SP600125 (Figure 3G-3H). These findings suggest that a high-level UA condition could enhance ROS and apoptosis in EPC, and also activate caspase 3 and 9 through JNK pathway.

### High levels of uric acid increase cellular senescence and reduce EPC migration and tube formation

**Figure 4:**
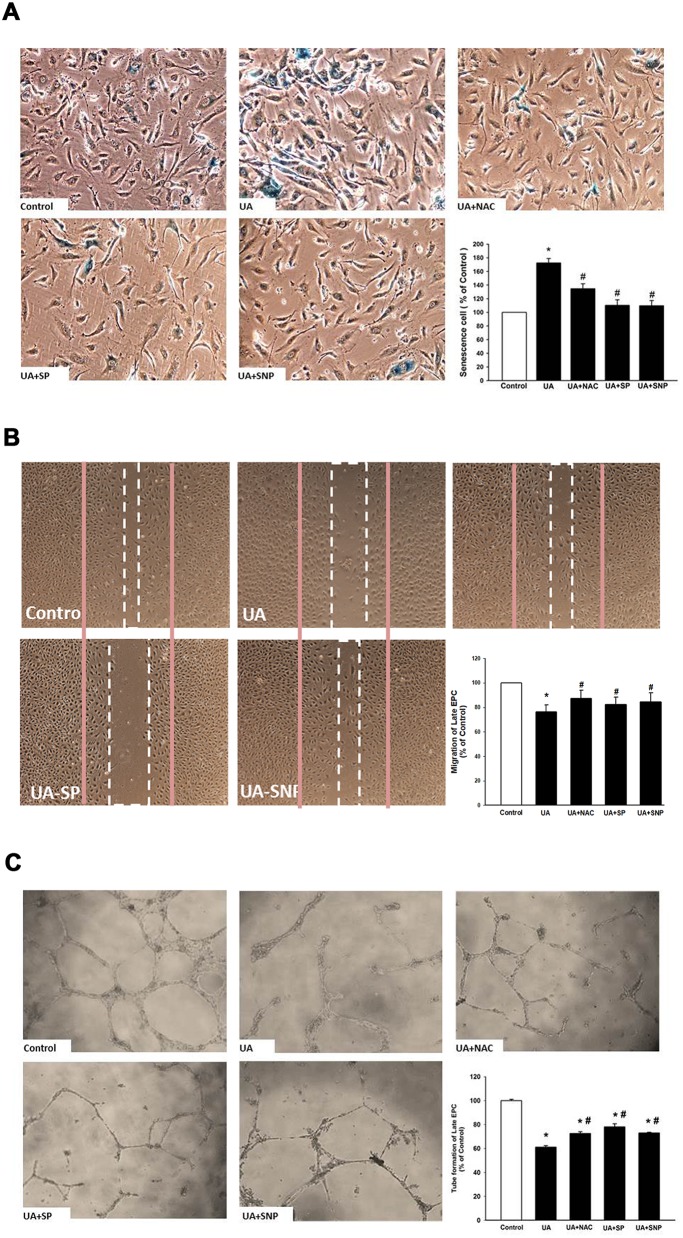
Effects of high-level uric acid on senescence, migration and angiogenic function of EPCCells were cultured at high-level uric acid concentration (10 mg/dL) in the absence or presence of SNP (NO donor), NAC (antioxidant) and SP (SP600125, JNK inhibitor) **(A)** Senescence of EPC was analyzed by senescence-associated acidic-β-galactosidase activity assay. **(B)** Migration of EPC was analyzed by scratch injury model. **(C)** Tube formation of EPC was analyzed with an angiogenesis assay kit. Data are mean ± SEM; n=6; ^*^ *P* < 0.05 versus control; ^#^ *P* < 0.05 versus high-level uric acid (10 mg/dL).

We further clarified the effect of high-level UA on senescence and the angiogenesis function of EPC. As shown in Figure 4A-4C, incubation of EPC in a high-level UA medium significantly enhanced EPC senescence and reduced EPC migration and tube formation. These effects could be partly reversed by administration of a NO-donor (SNP), antioxidant (NAC), and JNK inhibitor (SP600125). In addition, administration of SNP, NAC, and JNK inhibitor (SP600125) partly reversed cell viability (Supplementary Figure 2).

### Chronic hyperuricemia impairs ischemia-induced blood flow recovery and new vessels formation

**Figure 5:**
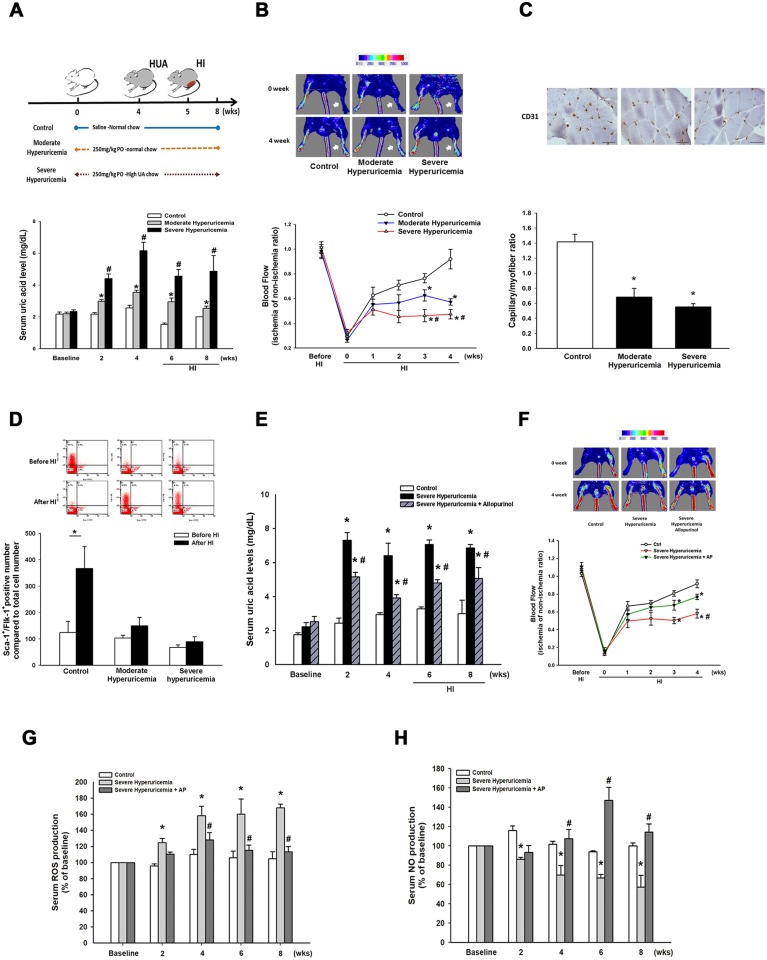
Blood perfusion and EPC mobilization in control and hyperuricemia mice after hindlimb surgery **(A)** Flow chart of hindlimb ischemia in the hyperuricemia model. Uric acid level in mouse serum was measured by automated clinical chemistry analyzer over time in the 3 groups (control, moderate and severe hyperuricemia groups). **(B)** Blood flow recovery was markedly impaired in the hyperuricemia mice as determined by laser Doppler imaging (n = 6 for each group) Quantification analysis of perfusion recovery by laser doppler perfusion imaging ratios (ischemic/normal hindlimb) over time in different groups. **(C)** Anti-CD31 immunostaining showed less development of collateral vessels and capillary formation in hyperuricemia mice. **(D)** Quantification of EPC-like cells (defined as Sca-1^+^/Flk-1^+^ cells) mobilization before and after hindlimb surgery was determined by flow cytometry. **(E)** Uric acid level serum in the severe hyperuricemia group by administration of allopurinol. **(F)** Blood flow recovery was improved in the severe hyperuricemia mice treated with allopurinol. Quantification analysis of perfusion recovery by laser doppler perfusion imaging ratios (ischemic/normal hindlimb) over time in different groups. **(G)** Mice serum ROS production, and **(H)** NO production in periphery blood were measured by assay kits, respectively. Results are mean ± SEM; n=6; ^*^ *P* < 0.05 versus control; ^#^ *P* < 0.05 versus moderate hyperuricemia; ^**^ *P* < 0.05 versus before HI; ^* #^ *P* < 0.05 versus control and moderate hyperuricemia before HI; Scale bar: 50 μm.

Mice were randomly assigned into 1 of the following 3 groups of different treatments: (1) control group; (2) moderate hyperuricemia group; (3) severe hyperuricemia group. The baseline level of UA in the control mice was 2.2±0.1 mg/dL. As shown in Figure 5A, moderate hyperuricemia was induced by administration of potassium oxonate; the serum UA gradually increased and was maintained for 8 weeks (peak level, 4^th^ week, 3.53±0.13 mg/dL). Severe hyperuricemia was created by combining potassium oxonate and a UA-enriched diet; the serum UA was elevated to a peak level at the 4^th^ week (6.16±0.52 mg/dL). There were no significant differences among the 3 groups in body weight, as well as in liver and renal functions before and 4 weeks after potassium oxonate treatment (Supplementary Table 1).

To further evaluate the angiogenic effect of high-level UA, we induced hindlimb ischemia by performing unilateral hindlimb ischemia surgery in the control group and in the hyperuricemia mice (n = 8 per group). As shown in Figure 5B, the moderate and severe hyperuricemia mice showed delayed blood flow recovery after ischemia surgery, compared to the control group.

Furthermore, tissue histological analysis revealed that the capillary density in the ischemic limb was significantly decreased in the hyperuricemia mice, compared with the control group (control vs. moderate hyperuricemia vs. severe hyperuricemia, 1.42±0.10 vs. 0.68±0.11 vs. 0.55±0.041; P < 0.05; (Figure 5C). These data indicated that blood flow recovery and vessel formation were impaired in the hyperuricemia mice exposed to tissue ischemia.

### High-level uric acid reduces endothelial progenitor cell mobilization

To investigate the effect of chronic hyperuricemia on EPC mobilization after tissue ischemia, circulating EPC numbers (defined by Sca-1^+^/Flk-1^+^ cells) were determined by flow cytometry. The number of EPC at baseline did not differ between the control and moderate hyperuricemia mice, but was significantly lower in the severe hyperuricemia mice (Figure 5D). EPC mobilization was enhanced after tissue ischemia in the control group (baseline vs. 3 day after operation: 196.6±40.1 vs. 504.8±69.4 MNCs, P = 0.012). However, impaired mobilization of the EPC in the peripheral blood was observed in the moderate and severe hyperuricemia mice after hindlimb ischemia surgery (moderate hyperuricemia, baseline vs. 3 days after operation: 172.9±30.3 vs. 266.4±51.5 MNCs, P = 0.234; severe hyperuricemia, baseline vs. 3 day after operation: 59.2±8.6 vs. 106±26.1 MNCs, P = 0.103). These data suggest that mobilization of EPC in response to hindlimb ischemia could be suppressed under a high-level UA condition.

### Allopurinol rescues blood flow recovery in severe hyperuricemia mice

To further determine the effect of decrease of uric acid level on blood flow recovery in ischemic hindlimbs, we treated severe hyperuricemia mice by administration of allopurinol (10 mg/dL). As shown in Figure 5E, administration of allopurinol significantly reduced uric acid levels compared to severe hyperuricemia mice. Allopurinol-treated severe hyperuricemia mice had significantly increased ischemic/nonischemic limb blood perfusion ratio compared to the control group (Figure 5F). Moreover, treatment with allopurinol attenuated ROS and enhanced NO levels (Figure 5G & 5H).

## DISCUSSION

The main findings of this study are that: 1) chronic hyperuricemia impaired the mobilization of EPC; 2) chronic hyperuricemia decreased angiogenesis and blood flow recovery of ischemic tissues; 3) high-level UA induced cellular apoptosis and impaired angiogenic functions of EPC; 4) increased oxidative stress and reduced NO synthesis may be responsible for the noxious effect of high-level UA on EPC. Our results suggest the existence of a novel pathogenic mechanism that explains the detrimental effects of chronic hyperuricemia on cardiovascular systems.

Many epidemiological studies indicate that hyperuricemia is associated with an increased risk of cardiovascular disease [9, 20]. Nonetheless, the pathogenesis of UA-related vasculopathy remains to be determined. Accumulating evidence has suggested that endothelial dysfunction plays a central role in the development of UA related vascular pathology, because high-level UA initiates oxidative stress and reduces NO synthesis [21, 22]. Endothelial dysfunction manifests itself in a loss of balance between injury and repair capacity. EPC are bone marrow-derived stem cells that differentiate into endothelial cells. It is well appreciated that these progenitor cells are able to repair endothelium of injured vessels and enhance neovascularization of ischemic tissues [23]. Experimental studies and pilot clinical trials show that application of EPC therapeutically improves neovascularization after ischemia [24, 25]. Reduced numbers of EPC are also associated with endothelial dysfunction and risk of cardiovascular events [26]. However, the molecular mechanism underlying EPC and vascular events related to hyperuricemia remain largely unknown.

Angiogenesis is the sprouting of new blood vessels from pre-existing vascular structures, a physiological process in which tissues cope with ischemia. This process starts with degradation of collagen fibers in the basement membrane, followed by migration and proliferation of pre-existing vascular endothelial cells [25]. Recent evidence suggests that the incorporation of circulating EPC into endothelial cells *in situ* plays an important role in neovascularization [14]. In our animal studies, we demonstrated a significant mobilization of EPC after hindlimb surgery. Proper mobilization, retention and survival of EPC within the target tissue are also crucial for neovascularization. A previous study has shown that an acute surge of UA was a herald of tissue injury, which accelerated the recruitment of EPC [18]. In contrast, mobilization of EPC was blunted in animals with chronic hyperuricemia [18]. In line with previous findings, we found a blunted mobilization of EPC after hindlimb surgery in chronic hyperuricemia mice. Furthermore, neovascularization of ischemic hindlimb was compromised with impairment of reperfusion. These pathological and functional effects were consistent with the inhibitory effect of UA on EPC in cultures. The concentrations that were used in cultured EPC were comparable to those in health and hyperuricemic patients (5 mg/dl and 10 mg/dl), suggesting these *in vitro* effects may be translated into *in vivo* situations. To the best of our knowledge, ours was the first demonstration of the suppressive effect of UA on neovascularization and blood flow recovery. Our results provide a new link between UA and vascular diseases.

Previous studies have focused on the effect of UA on mature vascular endothelial cells. Most studies show that the entry of UA into endothelial cells is associated with a reduction in NO bioavailability via a variety of mechanisms, including blocking uptake of L-arginine, stimulating L-arginine degradation and by the presence of UA itself [22, 27, 28]. Compared to mature endothelial cells, EPC were shown to express lower levels of eNOS [29, 30]. The effects of UA on progenitor cells remain unknown. In our study, high concentrations of UA down-regulated NO production in cultured EPC. Phosphorylated eNOS protein was decreased in the high-level UA group, as was the phosphorylated serine/threonine protein kinase Akt [31]. Our results suggest that high-level UA may attenuate the production of NO via the Akt-eNOS-NO pathway in EPC, and that NO-donor could partially reverse this effect. In mature endothelial cells, expression and phosphorylation of eNOS are known to be essential for the survival, migration, and angiogenic function of EPC, and NO promotes the mobilization of EPC from bone marrow [32–34]. All of this evidence supports our findings that NO plays a critical role in hyperuricemia-related impairment of neovascularization.

**Figure 6:**
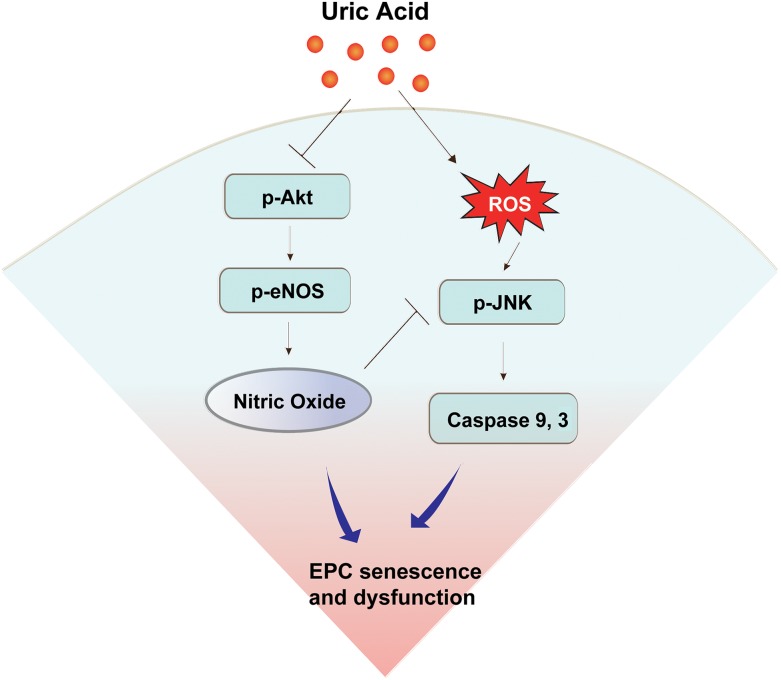
Schematic diagram summarizing possible mechanisms by which uric acid induces endothelial progenitor cell (EPC) damage Uric acid resulted in the impairment of EPC function by down-regulation of the Akt/eNOS/NO pathway and up-regulation of caspase 3 and caspase 9 through the JNK pathway.

Entry of UA into cells increases ROS [35–37]. Hyperuricemia could cause overproduction of ROS via activation of NADPH [37, 38]. Low levels of ROS regulate various signal transduction pathways involved in cell survival, differentiation and growth; high levels of ROS are harmful and have been implicated in the pathogenesis of vascular diseases [39]. EPC have low levels of ROS and reduced sensitivity to ROS-induced cell apoptosis [40]. We found that UA increased the formation of ROS in EPC in a dose-dependent manner, and that supplementing an anti-oxidant partially reversed the effect of ROS on the senescence and angiogenic function of EPC. Oxidative stress-induced activation of JNK signaling is an important step in the induction of cell apoptosis [41]. When the formation of ROS exceeds the capacity of the intracellular antioxidative defense system, the JNK pathway is activated, and its activation eventually leads to apoptosis [42]. Our results revealed an up-regulation of JNK phosphorylation in cultured EPC treated with high-level UA, and JNK inhibitor reversed this effect. Caspases are a family of protease enzymes that play crucial roles in apoptosis [43]. In cultured EPC treated with UA, the expressions of both caspase 3, an executional caspase, and caspase 9, an initiation caspase, are up-regulated in a dose-dependent manner. The upregulation of caspase 3 and 9 could be reversed by administration of a NO-donor, anti-oxidant or JNK inhibitor. Taking all these findings together, UA may induce apoptosis and impair the angiogenic function of EPC through ROS production and NO deprivation. The mechanisms of UA-induced EPC damage were summarized in Figure 6.

In conclusion, our findings suggest that neovascularization and blood flow recovery was related to EPC, and was impaired in a chronic hyperuricemic status. UA impaired the proliferation and angiogenic functional competence of EPC. Both ROS and NO may mediate the uric acid-related EPC dysfunction. These findings not only provide insights into the complex mechanism of vascular events related to UA, but also provide a potential therapeutic target to improve vascular competence after ischemic events or revascularization.

## MATERIALS AND METHODS

### Cell cultivation and reagent

Total mononuclear cells (MNCs) were isolated by density gradient centrifugation with Histopaque-1077 (1.077 g/mL, Sigma) from peripheral blood of healthy young volunteers. In brief, 5 × 10^6^ MNCs were seeded on a 0.1% fibronectin-coated plate in EGM-2MV medium (Cambrex, East Rutherford, NJ, USA), with supplements (hydrocortisone, human epidermal growth factor, human fibroblast growth factor, R^3^-insulin-like growth factor 1, vascular endothelial growth factor, gentamicin, amphotericin B, vitamin C, and 20% fetal bovine serum) at 37°C in a 5% CO_2_ incubator. The medium was changed and non-adherent cells were removed after every 4 days of culture. EPC appeared within 7-15 days after the start of the MNC culture. Furthermore, we identified EPC by the cell biomarkers CD31, CD34, CD133, KDR (Santa Cruz), and vWF (Neomarkers) using immunofluorescence. The cell images were obtained by laser scanning confocal microscope and the quantitative analysis by the Metamorph Premier Offline software measured. UA (Sigma, St. Louis, MO, USA) was dissolved in 0.01M NaOH to a final concentration of 50mM and then diluted in culture medium. All cell experiments used passage numbers 3 through 7.

### EPC viability

After the cells had been treated with UA in different concentrations ranging from 5-20mg/dL and for different periods ranging from 0-24 hours, EPC were treated with 3-(4,5-dimethylthiazol-2-yl)-2,5,diphenyltetrazolium bromide (MTT, 0.5 mg/mL, Sigma) for 4 hours, lysed with dimethyl sulfoxide, and their absorbance was measured at 550/650 nm.

### EPC senescence

Cellular aging was determined with a Senescence Cell Staining kit (Sigma). Briefly, after washing with phosphate-buffered saline (PBS), EPC were fixed for 6 minutes in 2% formaldehyde and 0.2% glutaraldehyde in PBS, and then incubated for 12 hours at 37°C without CO_2_ and with fresh X-gal staining solution (1 mg/ml X-gal, 5 mM potassium ferricyanide, and 2 mM MgCl_2_; pH6). After staining, green-stained cells and total cells were counted and the percentage of β-galactosidase-positive cells was calculated [44].

### Scratch injury model in EPC

The migratory function of EPCs was evaluated by a scratch injury model. EPCs were seeded onto 6-well cell culture plates. Once at the confluence, cells were treated with uric acid for 24 hours. And then scrape the cell monolayer in a straight line to create a scratch with a p200 pipette tip. After an injury, the monolayer was gently washed with PBS, and the medium was replaced with medium containing 10% FBS. EPCs sprouting from the edge of the injured monolayer was examined and photographed before and at 16 hours after scratching. Migrated endothelial cells were counted in 6 randomly selected high-power fields adjacent to the scratch injury.

### EPC tube formation

EPC tube formation is another essential function of vasculogenesis. Cell tube formation was performed with an *In Vitro* Angiogenesis Assay Kit (Chemicon). The protocol followed the manufacturer’s instructions. ECMatrix gel solution was thawed at 4°C overnight and mixed with ECMatrix diluent buffer. The gel was placed in a 96-well plate at 37°C in a 5% CO_2_ incubator for 1 hour. 1× 10^4^ EPC were seeded in matrix solution and incubated at 37°C in a 5% CO_2_ incubator for 16 hours. Then, cells were stained using *a*cetylated-low density lipoprotein (Ac-LDL). Cell tubule formation was inspected under a fluorescence microscope. Six random representative fields were taken, and the average of the total area of complete tubes formed by cells was compared by using computer software, Image-Pro Plus.

### Nitric oxide and reactive oxygen species production measurement

3-amino, 4-aminomethyl-2’, 7’-difluorofluorescein (DAF-FM) diacetate (10 μM, Molecular Probes) was used to determine nitric oxide (NO) by staining the cells for 30 minutes. The cells were lysed using lysis buffer and then the fluorescence intensity (relative fluorescence units) was evaluated at excitation and emission wave length 495/515-nm using a fluorescence microplate reader.

2’, 7’- dichlorodihydrofluorescein diacetate (DCFH-DA 20 μM, Molecular Probes) was used to determine a kind of reactive oxygen species (ROS), H_2_O_2_, by staining the cells for 30 minutes. The cell were lysed using lysis buffer and then the fluorescence intensity (relative fluorescence units) was evaluated at excitation and emission wavelength 485/530-nm using a fluorescence microplate reader.

### Annexin V analysis

Apoptosis was detected using the Annexin V FITC Assay (BD Pharmingen^TM^) and the Cytomics FC 500 (Beckman Coulter, Marseille, France). The measuring protocol was according to the manufacturer’s instructions. In brief, cells were washed with cold PBS twice and then the cells were resuspended in Binding Buffer at a concentration of 1×10^6^ cells/ml. The cells were stained using FITC Annexin V and PI dye, incubated for 15 minutes at room temperature in the dark, and analyzed by flow cytometry within 1 hour.

### Western blot analysis

First, EPC protein was acquired and lysed in protein lysis buffer (62.5 mM Tris-HCl, 2% SDS, 10% glycerol, 1 mM PMSF, and protein inhibitor including 1 μg/mL aprotinin, pepstatin, and leupeptin). The protein was subjected to SDS-PAGE by gel electrophoresis, followed by electro-blotting onto a PVDF membrane. Membranes were probed with antibodies against that directed to β-actin, SirT1 (Cell Signaling, Danvers, MA, USA), p16 (Abcam, Cambridge, MA, USA), phosphorylated eNOS (p-eNOS), eNOS (Millipore, Billerica, MA, USA), phosphorylated Akt (p-Akt), Akt (Cell Signaling, Danvers, MA, USA), phosphorylated ERK (pERK), ERK, and activated-caspase 3,9 (Cell Signaling, Danvers, MA, USA). The protein blots were detected by chemi-luminescence using ImageQuant LAS 400.

### Animals

Male C57BL/6J mice with a body weight of about 24 g (6 weeks old) were purchased from the National Laboratory Animal Center, Taiwan. The animal study protocol conformed to the Guide for the Care and Use of Laboratory Animals published by the US National Institutes of Health (NIH Publication 1996). The animal study received the approval of the Institutional Animal Care Committee of National Yang-Ming University (Taipei, Taiwan). All of the mice were given a standard laboratory diet and water ad libitum. Moreover, they were kept in micro-isolator cages on a 12-h day/night cycle.

### Hyperuricemia model

Potassium oxonate is an uricase inhibitor (Stavric et al., 1995), and was used to induce hyperuricemia in the mice. The mice were fed a UA-enriched diet (normal diet containing 5% potassium oxonate and 2.5% UA), and were randomly assigned into 1 of the following 4 groups involving different treatments: (1) vehicle group (mice treated with saline of the same volume); (2) potassium oxonate group (moderate hyperuricemia group); (3) potassium oxonate + high-level UA diet group (severe hyperuricemia group); (4) potassium oxonate + high-level UA diet group + Allopurinol (severe hyperuricemia + Allopurinol group). Potassium oxonate was given by an intraperitoneal injection at a dosage of 250 mg/kg. And, Allopurinol was given by a gavage feeding at a dosage of 10 mg/kg. Body weight, serum UA, creatinine (Cr), blood urea nitrogen (BUN), Glutamic Oxaloacetic Transaminase (GOT) and Glutamic Pyruvic Transaminase (GPT) were measured every 2 weeks during the experiment period.

### Mouse hindlimb ischemic model

Excision of the right femoral artery resulted in unilateral hindlimb ischemia, as previously described. The animals were anesthetized by intraperitoneal injection of tribromoethanol (Avertin) (250 mg/kg) before surgery. The proximal and distal portions of the right femoral artery were ligated. Hindlimb blood perfusion was measured using a laser Doppler Perfusion Imager (LDPI) System (Moor Instruments Limited, Devon, UK) before and after surgery, and then weekly. Then, the ratio of perfusion in the ischemic versus non-ischemic limb was measured.

### Measurement of serum levels of uric acid, creatinine, blood urea nitrogen, Glutamic Oxaloacetic Transaminase and Glutamic Pyruvic Transaminase

We collected whole blood from the mice, and allowed it to stand at room temperature for 30 minutes. The serum was obtained by centrifugation at 3000×g for 15 minutes at 4°C, and kept at −80°C until analysis. Serum samples were measured using biochemical slides with an automated Clinical Chemistry Analyzer (FUJI DRI-CHEM 4000i).

### Reactive oxygen species measurement in mice

ROS was determined in peripheral blood in mice every 2 weeks. In brief, ROS was measured by chemiluminescence with a ROS assay kit (Sigma, St. Louis, MO, USA). The fluorescence intensity (relative fluorescence units) was evaluated at excitation and emission wavelength 640/675-nm using a fluorescence microplate reader.

### Nitric oxide measurement in mice

NO determined in peripheral blood in mice every 2 weeks and was measured by quantification of nitric oxide and nitrate/nitrite parameter assay kit following the manufacturer’s protocol (R&D Systems, Ltd., USA). No level was determined by the optical density (O.D.) of each well using a microplate reader set at 540 nm (wavelength correction at 690 nm).

### EPC mobilization in a hyperuricemia hindlimb ischemic model

We wanted to understand EPC mobilization in a hyperuricemia model in response to tissue ischemia (72 hours after surgery). We collected peripheral blood MNCs and examined them with a fluorescence-activated cell sorter (FACS Calibur, Beckman Cytomics FC 500). We used fluorescein isothiocyanate (FITC) anti-mouse Sca-1 (eBioscience, San Diego, CA, USA) and phycoerythrin (PE) anti-mouse Flk-1 (VEGFR-2, eBioscience) antibodies with fluorescein isothiocyanate (FITC). Circulating EPCs were quantified by enumerating Sca-1^+^/Flk-1^+^ cells, compared to total cell number.

### Measurement of capillary density in the ischemic leg

The mice were sacrificed by intraperitoneal injection of an overdose of avertin 5 weeks after surgery. Then, the whole limbs were fixed in methanol and 30% sucrose overnight. The femora were carefully removed, and the ischemic thigh muscles were embedded in paraffin. Next, we de-paraffinized sections (5 μm) and incubated them with rat-polyclonal antibody against murine CD31 (Abcam ab28364, USA). Antibody distribution was visualized using the avidin-biotin-complex technique and Vector Red chromogenic substrate (Vector Laboratories, Burlingame, CA, USA), followed by counterstaining with hematoxylin. Capillaries were identified by positive staining for CD31 and morphology. Capillary density was expressed as the number of capillaries per square millimeter.

### Statistical analysis

All quantitative data are presented as mean±SEM. Statistical analyses were performed using SPSS software (version 14; SPSS, Chicago, IL, USA). The unpaired Student’s *t*-test or variance analysis was used to evaluate comparisons between groups. A *P* value less than 0.05 was considered to be statistically significant.

## SUPPLEMENTARY MATERIALS FIGURES AND TABLE


