# Supplementary material for: Chronic hyperuricemia impairs blood flow recovery in the ischemic hindlimb through suppression of endothelial progenitor cells
Source: Oncotarget. 2018 Jan 22;9(10):9285–98. doi: 10.18632/oncotarget.24290 (PMC5823617; doi:10.18632/oncotarget.24290)
Supplement: Supplementary file 1 [file oncotarget-09-9285-s001.pdf]

Chronic hyperuricemia impairs blood flow recovery in the ischemic hindlimb through suppression of endothelial progenitor cells

SUPPLEMENTARY MATERIALS

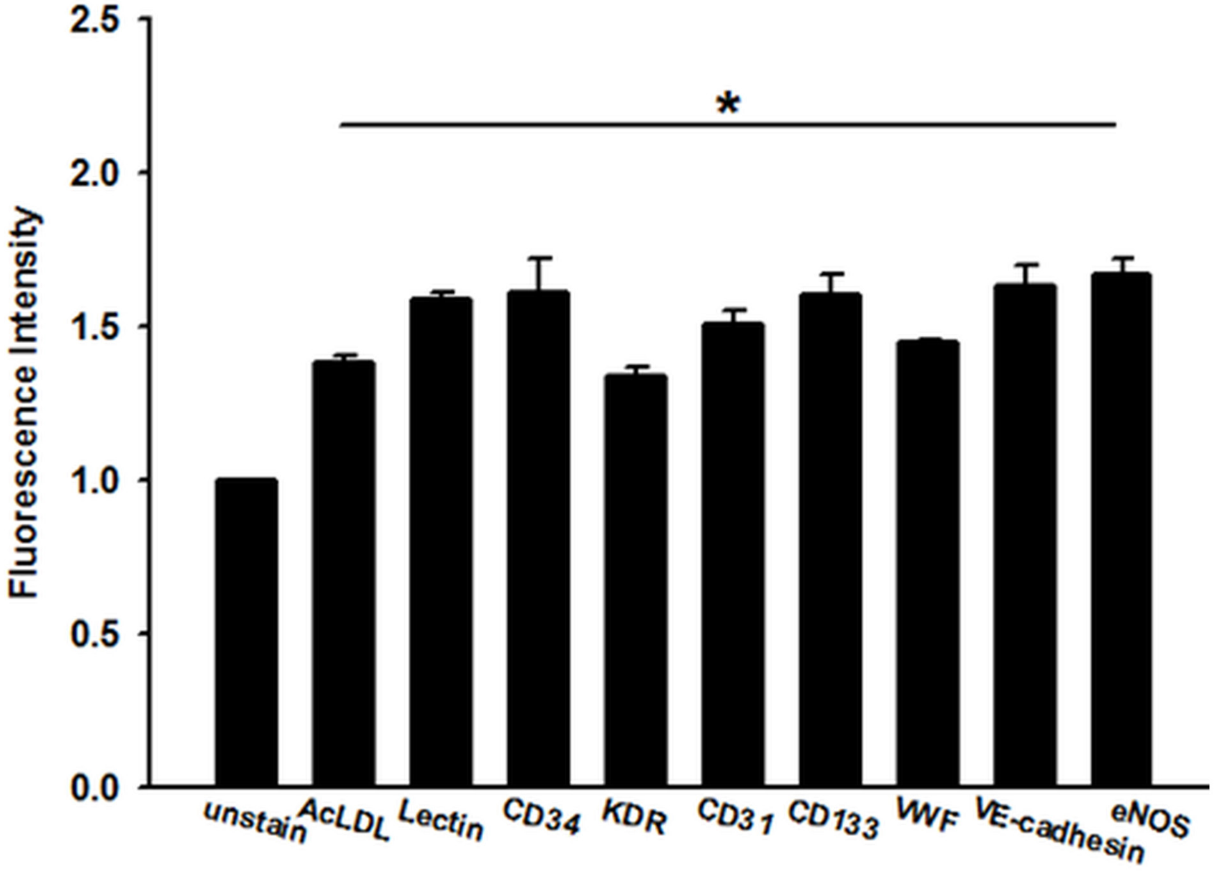

Supplementary Figure 1: Quantitative data of the cultured EPC markers.

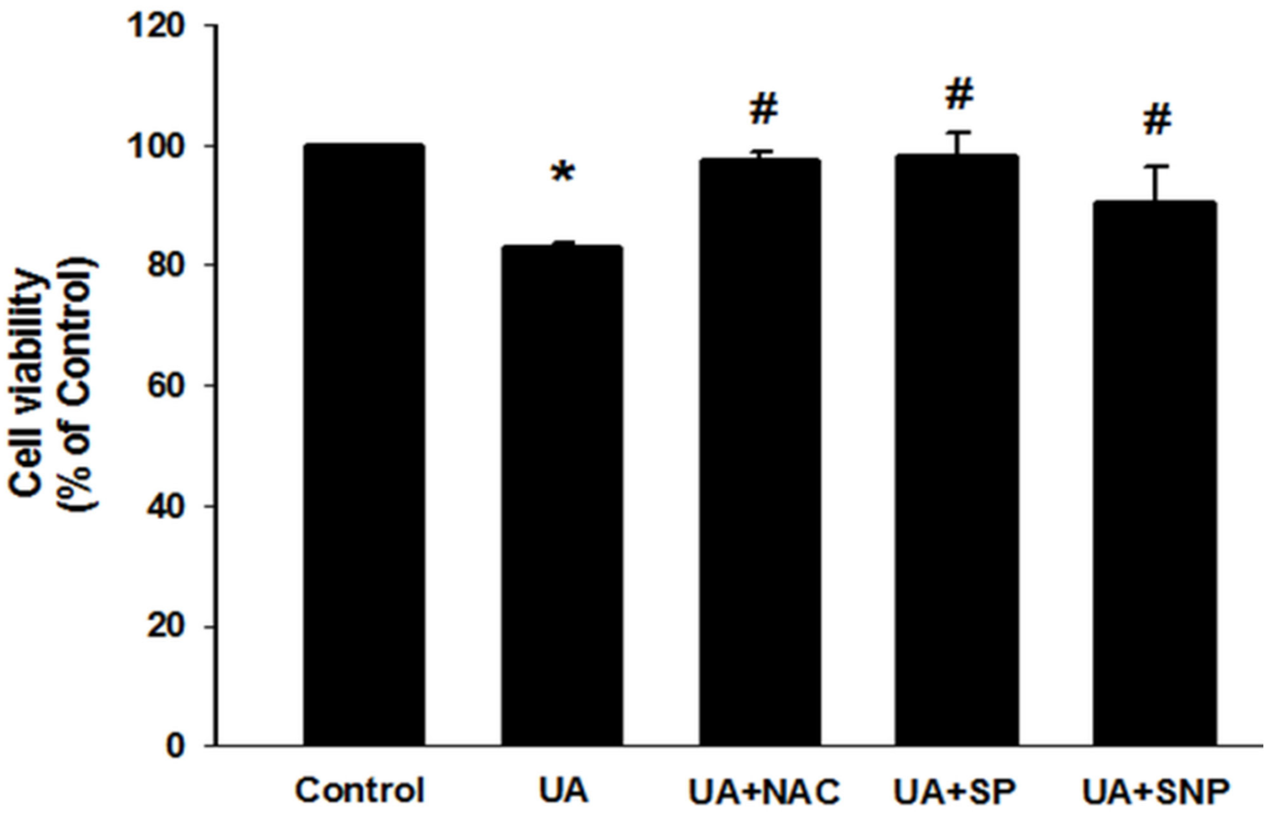

Supplementary Figure 2: EPC viability was analyzed by MTT assay. EPC viability was compared when cultured at concentration of uric acid (10 mg/dL) for 24 hours and in the absence or presence of NAC (antioxidant), SP (SP600125, JNK inhibitor), and SNP (NO donor).

Supplementary Table 1: Body weight, plasma renal and liver functions at baseline and 4 weeks after treatment of study animals

	Control		Moderate Hyperuricemia		Severe Hyperuricemia		Severe Hyperuricemia allopurinol	
**Week**	**0**	**4**	**0**	**4**	**0**	**4**	**0**	**4**
**Body wight (g)**	24.2 ± 0.58	25.0 ± 0.63	24.0 ± 0.44	24.8 ± 0.83	24.0 ± 0.44	24.9 ± 0.37	23.6 ± 0.24	24.2 ± 0.2
**CRE (mg/dL)**	0.20 ± 0.05	0.33 ± 0.05	0.30 ± 0.03	0.20 ± 0.03	0.3 ± 0.04	0.2 ± 0.02	0.34 ± 0.02	0.26 ± 0.02
**BUN (mg/dL)**	30.93 ± 1.71	26.44 ± 1.45	28.82 ± 1.85	27.92 ± 0.79	29.88 ± 0.84	32.37 ± 3.01	24.62 ± 0.77	27.62 ± 1.98
**GOT (U/L)**	35.14 ± 2.01	25.14 ± 1.52	31.00 ± 1.67	31.50 ± 1.95	29.4 ± 1.97	32.50 ± 4.72	31.90 ± 1.53	33.60 ± 1.46
**GPT (U/L)**	10.00 ± 1.13	11.29 ± 1.04	11.17 ± 1.05	9.00 ± 0.76	12.00 ± 1.03	11.50 ± 2.47	11.85 ± 0.75	9.97 ± 0.88
